# Platelet-Rich Plasma for the Treatment of Plaque Psoriasis: A Systematic Review

**DOI:** 10.7759/cureus.50356

**Published:** 2023-12-11

**Authors:** Armando Bunjaj, Lucas Brandao, Kelly Siracuse, Varun Soti

**Affiliations:** 1 Neurological Surgery, Lake Erie College of Osteopathic Medicine, Elmira, USA; 2 Internal Medicine, Lake Erie College of Osteopathic Medicine, Elmira, USA; 3 Dermatology, Lake Erie College of Osteopathic Medicine, Elmira, USA; 4 Pharmacology and Therapeutics, Lake Erie College of Osteopathic Medicine, Elmira, USA

**Keywords:** interleukin 17, prp, platelet-rich plasma, chronic plaque psoriasis, psoriasis treatment, plaque clearance, clinical benefit, plaque psoriasis, psoriasis

## Abstract

Psoriasis is a chronic and recurring condition characterized by scaly red plaques. The most common variant, plaque-type psoriasis, presents distinct clinical features. It profoundly impacts psychological and mental well-being, resulting in depression, anxiety, and suicidal thoughts. Psoriasis occurs due to disruptions in the skin’s innate and adaptive immune response triggered by trauma, infection, or medications. Treatment options include topical therapies such as corticosteroids and vitamin D analogs, phototherapy, conventional systemic agents such as methotrexate (MTX), and biologics that target pro-inflammatory cytokines. There has been growing interest in platelet-rich plasma (PRP) as a potential treatment option for plaque psoriasis, given its lower toxicity compared to existing approaches. However, its use is not yet widespread in clinical practice due to the limited awareness of its effectiveness. This review aims to investigate the efficacy of PRP therapy for plaque psoriasis. To conduct a comprehensive analysis, we followed the Preferred Reporting Items for Systematic Reviews and Meta-Analyses (PRISMA) guidelines, thoroughly searching PubMed, Elton Bryson Stephens Company (EBSCO), and ClinicalTrials.gov between February and July 2023. Our focus was on patients diagnosed with plaque psoriasis, and we found multiple studies that demonstrated promising results of PRP either as monotherapy or in combination with current treatments such as MTX. The clinical evidence strongly supports the effectiveness of PRP in treating plaque psoriasis. PRP significantly improves dermatological symptoms and enhances patient and physician satisfaction. Research suggests that PRP reduces the expression of interleukin (IL) 17, a pro-inflammatory mediator, explaining its mechanism of action in treating plaque psoriasis. However, additional clinical trials with larger sample sizes, including PRP as a separate treatment group and comparisons with positive and control groups, are necessary to reinforce its efficacy in plaque psoriasis patients and elucidate other potential mechanisms underlying its beneficial effects.

## Introduction and background

Psoriasis is a chronic, recurring, immune-mediated papulosquamous condition. It is characterized by scaly red plaques on the skin surface. The incidence of psoriasis varies globally, affecting approximately 3% of the population in the United States [1]. Psoriasis has two onset peaks, the first occurring between the ages of 15 and 20 and the second occurring between 55 and 60 [2]. A common form of psoriasis is plaque-type psoriasis, also known as psoriasis vulgaris. It is the most prevalent variant and presents distinct clinical features. These features include well-defined, red, itchy patches covered with silvery scales. The plaques can merge and spread across large areas of the skin, frequently affecting the trunk, limbs, and scalp [3].

Psoriasis significantly impacts a patient’s quality of life and overall well-being. Its implications on psychological and mental health are crucial, as it is associated with a higher prevalence of depression, anxiety, and suicidal thoughts. Psoriatic inflammation is driven by disturbances in the skin’s innate and adaptive immune responses [4]. In some patients, endogenous signals and cytokines activate the innate immune system, perpetuating autoinflammatory responses. In others, T cells play a role in autoimmune reactions. Therefore, psoriasis exhibits characteristics of both an autoimmune disease and an autoinflammatory condition, with overlapping mechanisms that potentially amplify each other [5].

Psoriasis primarily manifests on the outermost layer of the skin, made up of keratinocytes. However, the inflammatory effects of psoriatic plaques extend beyond the epidermis. The pathogenesis of psoriasis can be divided into initiation and maintenance. Trauma, infection, or drugs may trigger the initiation phase, while the maintenance phase is characterized by chronic progression. The exact mechanism of dendritic cells, which play a crucial role in the early stages of the disease, remains unclear. One proposed mechanism is the recognition of antimicrobial peptides (AMPs) released by injured keratinocytes, which are overexpressed in psoriatic skin. The maintenance phase of psoriatic inflammation occurs due to the activation of the adaptive immune response, primarily by distinct subsets of T cells. T helper 17 (Th17) cytokines, such as interleukin (IL) 17, IL-21, and IL-22, promote the proliferation of keratinocytes in the epidermis. Plaque-type psoriasis is characterized by the tumor necrosis factor-alpha (TNF-α)-IL-23-Th17 inflammatory pathway. Medications targeting TNF-α, IL-23, IL-17, and signaling pathways such as Janus kinase/signal transducer and activator of transcription (JAK/STAT) have proven effective in managing plaque psoriasis. However, these medications unfortunately come with multiple side effects [5].

The standard treatment options for psoriasis include topical therapy, phototherapy, and systemic therapy. Topical treatments include vitamin D analogs (calcipotriol) and corticosteroids, considered first-line treatments. Second-line treatments comprise phototherapy, psoralen with ultraviolet A radiation, and conventional systemic agents, such as methotrexate (MTX). Biological therapies may be considered for patients who do not respond to first- and second-line treatments [6]. MTX is a well-established drug used for the systemic treatment of chronic plaque psoriasis, receiving its first approval in 1972. However, approximately 78% of patients experience adverse reactions to MTX, with the most common complaints being nausea and vomiting when taken orally [7]. Severe adverse reactions, including life-threatening infections and death, have been reported with MTX therapy, according to the Food and Drug Administration (FDA) [8].

Extensive efforts have been made to develop innovative therapies for treating plaque psoriasis. This is due to the increased risk profile and potential toxicity of MTX. One such approach is the development of biologics, which are human monoclonal antibodies that target pro-inflammatory cytokines. These biologics have been shown to profoundly impact the outcomes of patients with moderate-to-severe psoriasis [9]. Over the past two decades, there has been a rapid evolution in the field of biologics. A study analyzing drug approval trends over the years indicated that 15.56% of drugs approved from 2009 to 2017 were biologics, signifying a rise in their development, FDA approval, and utilization for psoriasis treatment [10]. Noteworthy biologics recently approved for treating plaque psoriasis include risankizumab in 2019 and tildrakizumab in 2018 [11].

Despite the promising nature of biologics, their use has certain drawbacks. Regular subcutaneous or intravenous injections are required, and potential side effects can harm the body. Clinical trials have reported *Candida albicans* mucocutaneous infections, upper respiratory tract infections, and gastrointestinal disorders associated with biologics [12]. Many patients express discontentment with the adverse effects and contraindications of these biologics. In fact, a significant proportion of patients with moderate or severe psoriasis are dissatisfied with their current treatments [13].

Another prospective treatment option that has recently gained attention for plaque psoriasis is platelet-rich plasma (PRP). This treatment has been extensively studied for orthopedic injuries and dental complications, and it has also shown promise in aesthetic dermatology and wound healing. PRP has been introduced in clinical dermatology, with some studies demonstrating its potential to reduce the size of psoriatic plaques. The proposed PRP action mechanism involves inhibiting the nuclear factor kappa-light-chain-enhancer of activated B cells (NF-kB) and releasing growth factors from platelets [14-18].

Although PRP is considered a potential treatment option, it is currently outside the standard, routine clinical practice for treating psoriasis patients due to a lack of awareness regarding its effectiveness in treating plaque psoriasis. This review aims to evaluate and emphasize the progress made in using PRP as a therapeutic regimen for treating plaque psoriasis. Additionally, we seek to demonstrate the existing clinical evidence of the safety and effectiveness of utilizing PRP for this condition. Lastly, we aim to generate interest in using PRP to treat plaque psoriasis by highlighting the need for further investigation and posing unanswered questions.

## Review

Literature search and study selection

We conducted a comprehensive literature search, following the Preferred Reporting Items for Systematic Reviews and Meta-Analyses (PRISMA) guidelines [19], from February 2023 to July 2023. This involved searching PubMed and Elton Bryson Stephens Company (EBSCO) databases and exploring the ClinicalTrials.gov registry. The keywords used in the search were “platelet-rich plasma + psoriasis.”

The studies included various types of research, such as randomized controlled trials, non-randomized controlled trials, prospective investigations, pilot trials, retrospective studies, descriptive case series, case reports, and short reports. We specifically focused on patients diagnosed with psoriasis. Additionally, we found a relevant case report on a patient with trachyonychia, as it can be associated with psoriasis. We selected studies written in English, and if an investigation was originally published in a different language but subsequently translated into English and met the inclusion criteria, we included it in our review (see Table 1).

**Table 1:** Study selection criteria. In our review, we included studies published in English from 1980 to 2023, which focused on patients with psoriasis and met the inclusion criteria.

Inclusion criteria	Exclusion criteria
Randomized controlled trials	Preclinical studies
Non-randomized controlled trials	Systematic reviews
Prospective studies	Meta-analysis
Pilot studies	Narrative reviews
Retrospective studies	Commentaries
Descriptive case series	
Case report	
Short report	

**Figure 1:**
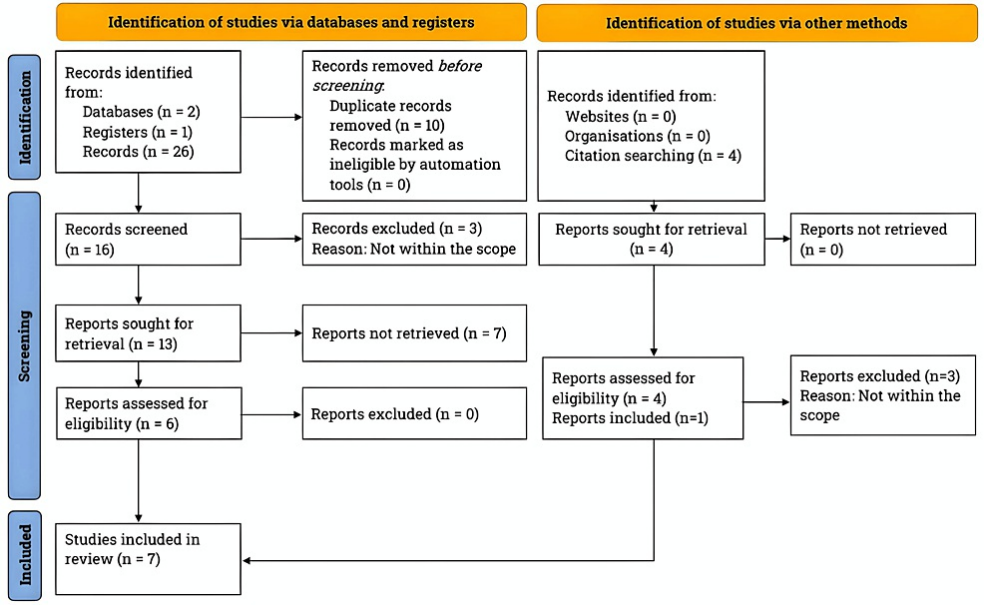
Literature search and study selection process. We adhered to the guidelines of the Preferred Reporting Items for Systematic Reviews and Meta-Analyses. We conducted a thorough search for clinical studies on the use of PRP in plaque psoriasis treatment using PubMed, EBSCO, and ClinicalTrials.gov. The search was performed between February and July 2023, and we specifically focused on studies published between 1980 and 2023. EBSCO, Elton Bryson Stephens Company; PRP, platelet-rich plasma

To evaluate the clinical evidence of each study, we assigned a specific level based on existing literature [20]. The PRISMA flowchart in Figure 1 outlines the study selection process and the total number of studies reviewed for this article.

Platelet-rich plasma

PRP refers to a fractionated plasma volume obtained from the patient’s blood, which is then concentrated with platelets. These platelets contain alpha granules rich in various growth factors such as platelet-derived growth factor, transforming growth factor-beta, insulin-like growth factor, vascular endothelial growth factor, and epidermal growth factor. These growth factors play a crucial role in the process of tissue repair. Medical professionals collect blood samples from the patient and use a centrifuge to generate PRP. The centrifugation separates the platelets and concentrates them within the plasma while separating the other blood components. Subsequently, the resulting PRP solution is typically injected directly into the affected area as a treatment [21].

Ongoing research aims to determine the optimal concentration, preparation methods, and timing for PRP injections. Various techniques and commercially available products exist, but their application can be perplexing due to the potential variation in the biological effects and the effectiveness of the resulting PRP products. Classification systems for PRP may consider factors such as platelet concentration, fibrin concentration, the presence of leukocytes, and activation status [21]. PRP injections are generally considered safe with minimal risks and typically do not result in significant side effects. Using one’s cells and plasma in PRP injections significantly reduces the likelihood of an allergic reaction compared to other injectable medications such as corticosteroids [22].

Autologous PRP injections were first documented in 1987 during open heart surgery [23]. Since then, PRP has been widely utilized in various medical fields. Over 20 years ago, dentistry utilized it to facilitate faster wound healing in cancer patients undergoing jaw reconstruction. Physicians have also used PRP to heal bones after spinal injuries and promote soft tissue recovery following plastic surgery [21]. In 2009, the FDA approved obtaining PRP and its use in orthopedics. However, it is still considered off-label for hair and skin procedures [24].

PRP treatment involves modifying the ratio of red blood cells to platelets to enhance the healing process. By reducing the content of red blood cells to 5% (since they offer less benefit for healing) and increasing the concentration of platelets to 94%, PRP becomes enriched with a powerful combination of growth factors. An average healthy individual typically has a platelet count between 150,000 and 450,000 cells per microliter of blood [21]. Studies have shown that the reliable enhancement of wound healing occurs when the platelet concentration is at least five times higher than average (approximately one million platelets/μL), while significantly higher concentrations do not provide further improvements in wound healing [25]. Although the precise mechanism of action remains uncertain, chronic inflammation is considered a potential pathway. In light of our understanding of psoriasis, PRP could be viewed as a viable treatment option that extends beyond orthopedics alone, and other avenues should be explored.

Clinical evidence of PRP’s effectiveness in treating plaque psoriasis

Kauhl et al. (2021) were among the most recent researchers investigating whether PRP can alleviate the burden of psoriasis. In a retrospective study involving 40 patients, the researchers examined the clinical effects of PRP on the skin of patients with plaque psoriasis or atopic dermatitis. Of the 40 patients, 30 were diagnosed with plaque psoriasis and received a subdermal injection of 5-6 mL of PRP after preparation with the autologous-conditioned plasma double syringe. The researchers calculated lesion size, Psoriasis Area and Severity Index (PASI), and Eczema Area and Severity Index at follow-up intervals (three, six, nine, and 12 weeks) after treatment to assess overall improvement trends [26].

The findings revealed a significant decrease in the average lesion size, from 8.2 cm^2^ to 0.3 cm^2^ (p < 0.00001). Remarkably, 80% of patients treated for plaque psoriasis achieved complete remission 12 weeks after treatment, and no adverse events were reported. This indicates that PRP therapy effectively treats psoriasis without posing substantial health risks to the patient. However, the researchers acknowledged the limitations of their study, particularly the analysis of data in an unblinded setting, which might introduce bias. Furthermore, the study had a small sample size and a relatively short follow-up period and lacked a control group. Nonetheless, the analysis presented encouraging results regarding psoriasis treatment with PRP [26].

Anwar et al. (2022) supported PRP as an effective option for treating psoriasis. Their descriptive case series involved treating 73 patients with intralesional PRP and MTX in psoriatic plaques. The patient’s PASI score needed to be equal to or greater than 75% at the 16th week of treatment to demonstrate that PRP worked effectively. The results showed that 82.19% of the cases met the effectiveness criteria, while 17.81% did not. Additionally, the researchers compared the efficacy of PRP across different age groups and genders [27].

Regarding age, 45% of patients were within the 18-39 age range, while 55% were in the 40-60 age range. The difference in PRP’s efficacy between these age groups was not statistically significant (p = 0.279). With regard to gender, 28.3% of the cases were male, and 71.1% were female. The difference in effectiveness between genders was also not statistically significant (p = 0.076). These findings suggested that PRP, in combination with intralesional MTX, was highly beneficial for patients irrespective of age or gender. However, it was noteworthy that this descriptive case series did not include a control group, making it difficult to establish a cause-effect relationship. Despite these limitations, the study served as a foundation for future research exploring the individual roles of subcutaneous MTX and PRP in treating psoriasis while comparing them with combination therapy [27].

In a prospective study, Chakravdhanula et al. (2016) examined the effectiveness of combining PRP with MTX in treating chronic plaque psoriasis. They enrolled 21 patients with a minimum PASI score of 10. Out of these patients, 16 were assigned to receive the combination treatment (PRP and MTX), while the remaining five received monotherapy (MTX alone). They administered PRP and MTX through the intralesional route to avoid the toxicity linked to systemic administration. The researchers compared the reduction trends in erythema, induration, and desquamation using digital photography and the PASI score at zero, four, eight, 12, and 16 weeks [28].

The results indicated that the combination of PRP and MTX showed superior efficacy in managing hard-to-treat psoriasis, irrespective of PASI score, disease duration, or previous systemic treatment. Patients who received the combination treatment demonstrated noticeable improvement in reducing erythema, induration, and desquamation at each follow-up visit, which was significant compared to those who received only MTX. A higher percentage of patients achieved remarkable clearance from psoriasis. By week 16, all patients in the combination therapy group achieved a 50% reduction in their PASI score, with 10 out of 16 (62.5%) achieving a 75% reduction and two out of 16 (12.5%) achieving a 90% reduction. In contrast, none of the patients in the monotherapy group achieved a 50% reduction in their PASI score. However, they exhibited improvement in the 35%-40% range compared to their baseline PASI scores. Importantly, patients in the combination group tolerated the treatment well without any serious adverse events [28].

While the results yielded substantial patient improvement, it is essential to note that the study design lacked statistical analysis. The researchers solely reported outcomes in percentages and did not conduct any statistical tests to establish significance. Furthermore, the sample size was relatively small. However, they effectively showcased the potential reduction of plaque psoriasis by combining PRP and MTX instead of MTX alone. Additionally, they showed that treatment administration through an intralesional route could prevent systemic adverse effects associated with the treatment [28].

Rahman et al. (2018) conducted an extensive study to comprehensively investigate PRP’s effects in treating chronic localized recalcitrant plaque psoriasis. The study also aimed to assess the satisfaction levels of patients and physicians with PRP treatment, making it the first study of its kind. The researchers recruited 30 patients with established chronic recalcitrant psoriasis in a localized area in a prospective controlled trial and assigned them to two groups. One group received intralesional PRP treatment, while the control group received emollient treatment. The study followed the patients for eight to 16 weeks, recording their PASI scores and satisfaction levels post treatment [29].

The study findings demonstrated that PASI scores significantly decreased after eight weeks (p = 0.001) and 16 weeks (p = 0.001) post-PRP treatment compared to baseline PASI scores. In contrast, there was no significant change in the PASI scores in the control group after eight weeks (p = 0.195) and 16 weeks (p = 0.894) compared to baseline. Also, the PASI scores were significantly reduced between eight and 16 weeks post-PRP treatment compared to baseline (p = 0.0018). However, there was no change in PASI scores in the control group compared to baseline during this duration (p = 0.121). Additionally, there was no incidence of adverse reactions to the PRP treatment. Regarding patient and physician satisfaction, patients in the PRP group exhibited significantly higher satisfaction levels with the treatment outcomes than those in the control group (p = 0.034). These findings were consistent with the satisfaction levels reported by physicians, who expressed a markedly higher satisfaction rate with patient outcomes in the PRP group than in the control group (p = 0.042) [29].

Kaur and Jakhar (2019) documented two cases of nail lichen striatus and trachyonychia. Both patients were unresponsive to topical steroidal treatment and instead responded well to PRP therapy. They received PRP injections into the nail matrix at three-week intervals. Weekly follow-ups were conducted for the initial three weeks, with subsequent assessments at 16 and 20 weeks. The progress of both patients was evaluated through photographic and dermoscopic examination. One patient demonstrated significant improvement within three weeks, while the other showed improvement within six weeks. No recurrences were observed during the 16- and 20-week follow-up period. Also noteworthy was the lack of any incidence of post-treatment adverse effects [30].

The researchers demonstrated the effective use of PRP in treating nail refractory disorders. However, they did not disclose other medical histories of the two patients or whether they had a prior history of psoriasis. Nevertheless, the study findings indicated that PRP could successfully treat nail lichen striatus and trachyonychia without any subsequent complications. However, additional studies on patients with nail lichen striatus, trachyonychia, and cutaneous manifestations of psoriasis involving larger sample sizes and appropriate controls must validate these results [30].

Multiple clinical studies have strongly indicated PRP therapy’s effectiveness in treating plaque psoriasis. However, only a few researchers have focused on the role of platelets in psoriasis and the mechanism of PRP in plaque psoriasis. One of the earlier attempts by Hayashi et al. (1985) investigated the involvement of platelet aggregation in the development of psoriasis. The experimental group consisted of 25 subjects with plaque psoriasis, and the control group included 50 normal subjects. The study aimed to assess and compare platelet aggregation in fasting PRP psoriatic subjects and normal subjects, using adenosine diphosphate (ADP), epinephrine (EPI), and collagen as platelet-aggregating agents [31].

The study findings revealed significantly higher platelet aggregation in psoriatic subjects compared to the control group. The maximum percentage of platelet aggregation (MPA) induced by ADP in psoriatic subjects was 47 ± 20, whereas in normal subjects, it was 30.6 ± 13.8 (p < 0.01). Similarly, MPA induced by EPI in psoriatic subjects was 37.5 ± 25.7 compared to 24.5 ± 23.5 in normal subjects (p < 0.05). MPA induced by collagen was 37.7 ± 31.8 in psoriatic subjects, while in normal subjects, it was 13.7 ± 21.7 (p < 0.001). These results suggested that the metabolism of arachidonic acid and metabolites, crucial to platelet aggregation, was dysregulated in psoriasis, contributing to hyper-aggregation of platelets in psoriasis [31].

These groundbreaking results laid the foundation for subsequent research, revealing the crucial role of bioactive lipid mediators derived from polyunsaturated fatty acids, such as arachidonic acid, in maintaining skin health. These mediators can have both anti-inflammatory and pro-inflammatory effects. An imbalance in these mediators, due to an inflammatory autoimmune response, can lead to changes in skin integrity and a range of skin diseases, including psoriasis [32].

Having recognized the role of inflammation in the development of plaque psoriasis [33] and considering various clinical studies demonstrating the efficacy of PRP in clearing psoriatic plaques, Mohammed et al. (2018) assessed the potential of PRP in regulating inflammatory molecules in psoriasis. Specifically, they aimed to elucidate the underlying mechanism of PRP concerning the expression of IL-17. In a randomized controlled clinical trial, they recruited 24 patients with plaque psoriasis and selected two symmetrical plaques per patient. One plaque received the intradermal administration of PRP, while the other received normal saline as a placebo. They performed biopsies before and after treatment to investigate the role of IL-17 expression in psoriasis pathogenesis and PRP’s mechanism [34].

The results of the study demonstrated that PRP treatment significantly reduced plaque size from 25.83 ± 29.02 cm (pre-PRP treatment) to 17.39 ± 20.77 cm (post-PRP treatment) (p < 0.001). However, saline treatment did not decrease plaque size (p = 0.287). Moreover, PRP treatment remarkably reduced plaque thickness from 2.58 ± 0.97 cm (pre-PRP treatment) to 1.71 ± 1.23 cm (post-PRP treatment) (p = 0.010). In comparison, saline treatment did not reduce plaque thickness (p = 0.073). PRP treatment and saline significantly reduced scaling (p = 0.007 and p = 0.013, respectively) [34].

Furthermore, pre-treatment biopsies revealed that 19 of 24 patients had IL-17 expression. Treatment with PRP resulted in the moderate-to-profound inhibition of IL-17 expression, with 10 out of 19 biopsies showing a moderate decrease in IL-17 expression and nine biopsies showing no IL-17 expression after PRP treatment compared to saline (p = 0.0171). The researchers demonstrated the effectiveness of PRP in treating plaque psoriasis and highlighted that PRP achieved this by reducing IL-17 expression in patients with plaque psoriasis. However, further research is needed to explore the relationship between PRP, IL-17 expression, and other inflammatory mediators in plaque psoriasis [34]. See Table 2 for the key studies and their findings reviewed in this paper.

**Table 2:** Key studies exploring the effectiveness of platelet-rich plasma (PRP) in treating plaque psoriasis. The table presents findings of the current literature on the effectiveness of PRP in treating patients with plaque psoriasis. Additionally, it includes studies highlighting platelets’ role in psoriasis pathogenesis, emphasizing their potential as a therapeutic target for PRP. Furthermore, it provides insights into PRP’s mechanism of action uncovered thus far. All the studies mentioned in the table meet the inclusion criteria of this systematic review.

Authors	Type of study	Sample size	Findings
Clinical evidence of the effectiveness of plasma-rich platelet (PRP) in treating plaque psoriasis			
Kauhl et al. (2021) [26]	Retrospective	40	PRP treatment significantly decreased the average lesion size, from 8.2 cm^2^ to 0.3 cm^2^ (p < 0.00001). Remarkably, 80% of patients treated for plaque psoriasis achieved complete remission 12 weeks post-PRP treatment. No adverse events were reported.
Anwar et al. (2022) [27]	Case series	73	Approximately 82.19% of the study’s patients demonstrated noteworthy improvement in their Psoriasis Area and Severity Index (PASI) score after 16 weeks of treatment with PRP. Both male and female patients aged 18-60 experienced effective results with PRP therapy. There were no statistical disparities in the benefits of PRP based on gender or age group.
Chakravdhanula et al. (2016) [28]	Non-randomized controlled trial	21	Patients who received a combination of PRP and methotrexate (MTX) experienced significant improvements in reducing erythema, induration, and desquamation compared to those who received MTX monotherapy. By week 16, all patients in the combination therapy group achieved a 50% reduction in their PASI score, with 10 out of 16 (62.5%) achieving a 75% reduction and two out of 16 (12.5%) achieving a 90% reduction. In contrast, no patient in the monotherapy group achieved a 50% reduction in their PASI score, and they only showed improvements in the 35%-40% range compared to their baseline PASI scores. Patients in the combination group tolerated the treatment well without experiencing any serious adverse events.
Rahman et al. (2018) [29]	Prospective controlled trial	30	PASI scores significantly decreased after eight weeks (p = 0.001) and 16 weeks (p = 0.001) post-PRP treatment, compared to the baseline scores. In contrast, the control group did not experience any significant change in PASI scores after eight and 16 weeks, in comparison to the baseline. Between eight and 16 weeks post-PRP treatment, there was a significant reduction in PASI scores compared to the baseline (p = 0.0018). Conversely, there was no change in PASI scores within the control group between eight and 16 weeks. Patients in the PRP group reported higher satisfaction levels with the treatment outcomes, compared to those in the control group (p = 0.034). Physicians also expressed a significantly higher satisfaction rate with patient outcomes in the PRP group, compared to the control group (p = 0.042). No adverse reactions were reported with the PRP treatment.
Kaur and Jakhar (2019) [30]	Case report	2	The patient with nail lichen striatus demonstrated significant improvement within three weeks of PRP treatment. The other patient with trachyonychia also showed remarkable improvement within six weeks of PRP treatment. Both patients were unresponsive to topical steroidal therapy. However, they responded to PRP therapy. There was no disease recurrence during the 16- and 20-week follow-up period post-PRP treatment. There was no incidence of post-treatment adverse effects.
Clinical evidence of the role of platelets and PRP’s mechanism in treating plaque psoriasis			
Hayashi et al. (1985) [31]	Randomized controlled trial	75	Psoriatic subjects exhibited significantly higher platelet aggregation compared to the control group. Adenosine diphosphate induced a maximum percentage of platelet aggregation (MPA) of 47 ± 20 in psoriatic subjects, whereas it was 30.6 ± 13.8 in normal subjects (p < 0.01). The MPA induced by epinephrine was 37.5 ± 25.7 in psoriatic subjects, in contrast to 24.5 ± 23.5 in normal subjects (p < 0.05). Conversely, the MPA induced by collagen was 37.7 ± 31.8 in psoriatic subjects and 13.7 ± 21.7 in normal subjects (p < 0.001). The hyper-aggregation of platelets contributed to psoriasis and could be a potential therapeutic target for PRP treatment.
Mohammed et al. (2018) [34]	Randomized controlled clinical trial	24	PRP treatment significantly reduced the size of the plaque from 25.83 ± 29.02 cm (pre-PRP treatment) to 17.39 ± 20.77 cm (post-PRP treatment) (p < 0.001). In contrast, saline treatment did not result in a decrease in plaque size (p = 0.287). Furthermore, PRP treatment remarkably reduced the thickness of the plaque from 2.58 ± 0.97 cm (pre-PRP treatment) to 1.71 ± 1.23 cm (post-PRP treatment) (p = 0.010). Interestingly, the saline treatment did not demonstrate any reduction in plaque thickness (p = 0.073). Both PRP treatment and saline significantly reduced scaling (p = 0.007 and p = 0.013, respectively). Notably, pre-treatment biopsies revealed that 19 out of 24 patients exhibited interleukin (IL) 17 expression. Treatment with PRP resulted in a moderate-to-profound inhibition of IL-17 expression. Ten out of 19 biopsies showed a moderate decrease in IL-17 expression, while nine biopsies demonstrated no IL-17 expression after PRP treatment in comparison to saline (p = 0.0171).

Discussion

PRP therapy has emerged as an efficacious and generally safe treatment option to treat plaque psoriasis patients. It has several advantages over traditional psoriasis treatments, such as MTX, corticosteroids, and biologics, such as minimal adverse effects. PRP utilizes the patient’s blood, reducing the risk of complications or severe side effects. However, whether PRP can reduce relapse rates typically associated with these conventional treatments in psoriatic patients remains to be determined [35].

Furthermore, as shown in the clinical evidence presented in this paper, PRP can substantially reduce PASI scores compared to other therapies. However, whether it can result in a remarkably enhanced quality of life for patients is yet to be studied. By decreasing the dependency on medications that carry the risk of severe side effects and providing a more innate approach to treatment, PRP has the potential to revolutionize the care for plaque psoriasis patients.

While several clinical studies have demonstrated the effectiveness of PRP therapy in treating plaque psoriasis, their smaller sample sizes have limited the overall understanding of the treatment’s impact and the generalization of the results. Although the existing research has shown promising results, the general consensus among medical researchers is that larger, more extensive studies are required [26-30,34]. By increasing the sample size, researchers can establish more substantial evidence of the effectiveness of PRP therapy for plaque psoriasis and further assess the potential benefits and risks associated with the treatment.

Future trials must ensure that PRP is utilized as a stand-alone treatment group and compared directly with positive and standard control groups. This will allow researchers to obtain a more precise effect of PRP and a better comparison with existing treatment options, such as MTX, corticosteroids, or biologics. Moreover, these studies should investigate various PRP preparation techniques and concentrations to determine the most effective protocols for administering this cutting-edge treatment to those suffering from the debilitating condition, plaque psoriasis.

As the clinical evidence becomes more decisive regarding the effectiveness of PRP in treating plaque psoriasis, there will be challenges associated with the accessibility and formulation of this treatment method. While commercial kits for PRP are available, formulating PRP requires specialized equipment and expertise, which may be limited in rural or remote areas. To overcome this challenge, collaboration between medical researchers and healthcare providers is crucial in developing simplified PRP preparation techniques suitable for smaller clinics and under-resourced medical facilities. Additionally, investing in training and educational programs for healthcare workers in rural and remote regions could significantly mitigate this issue [36].

The current management of plaque psoriasis involves oral medications, topical treatments, phototherapy, and biologics. However, these treatment options can lead to significant financial expenses for patients. The financial burden associated with these regimens is well-documented [37]. With ongoing research and advancements in PRP therapy, patients may experience long-lasting improvements that could potentially reduce or eliminate the need for other medications. As a result, the costs associated with treating their condition could be significantly reduced. Moreover, a well-executed PRP treatment might result in fewer follow-up and maintenance visits to healthcare providers, further easing the patient’s financial and logistical stresses. Despite these promising aspects, PRP’s impact on reducing healthcare costs in managing psoriasis is yet to be fully assessed.

## Conclusions

PRP therapy has emerged as an up-and-coming treatment option for plaque psoriasis. Clinical evidence strongly supports its efficacy in treating plaque psoriasis by reducing dermatological symptoms and improving patient satisfaction. Notably, existing research highlights PRP’s ability to reduce IL-17 expression in patients with plaque psoriasis, yielding clinical benefits. Moreover, further investigations are warranted to uncover other potential mechanisms underpinning PRP’s effectiveness and address challenges related to the accessibility and formulation of this therapy. Collaborative efforts between medical researchers and healthcare providers will be crucial in advancing PRP as a standardized treatment option, ultimately leading to cost reduction and enhanced quality of life for patients with plaque psoriasis.
